# Morning–evening differences of short-term maximal performance and psychological variables in female athletes

**DOI:** 10.3389/fphys.2024.1402147

**Published:** 2024-05-30

**Authors:** Wafa Jribi, Houda Bougrine, Ali Aloui, Jihen Khalfoun, Nafaa Souissi, Wajdi Mkacher, Kais El Abed, Abderraouf Ben Abderrahman

**Affiliations:** ^1^ High Institute of Sport and Physical Education Sfax, Sfax University, Sfax, Tunisia; ^2^ Education, High Institute of Sport and Physical Education of Sfax, University of Sfax, Sfax, Tunisia; ^3^ High Institute of Sport and Physical Education Gafsa, Gafsa University, Sfax, Tunisia; ^4^ Physical Activity Research Unit, Sport, and Health (UR18JS01), National Observatory of Sports, Tunis, Tunisia; ^5^ High Institute of Sport and Physical Education, Manouba University, Manouba, Tunisia; ^6^ Tunisian Research Laboratory “Sports Performance Optimization”, National Center of Medicine and Science in Sports (CNMSS), Tunis, Tunisia; ^7^ Department of Physiology and Lung Function Testing, Faculty of Medicine Ibn-El-Jazzar, University of Sousse, Sousse, Tunisia

**Keywords:** circadian rhythm, mood, physical performance, female athletes, psychological variables

## Abstract

The aim of this study was to examine the effect of time of day on short-term maximal performance and psychological variables in young females. Fifteen active women participated in the study (age: 22 ± 3 years) and completed Hooper and the POMS-F questionnaires subsequently. In a randomized order, they performed a maximum of 30 s cycling exercise at two different times of day: in the morning at 07:00 h and in the afternoon at 16:00 h with a recovery period of 48 h. The digit cancellation test, countermovement jump (CMJ), squat jump (SJ) test, and the lower quarter Y balance test were performed at the beginning and at the end of each session. Our results showed that only peak power and mean power (*p* < 0.01) during the maximum 30 s cycling, reaching distances during the Y-balance (*p* < 0.05), Jump height in CMJ and SJ (*p* < 0.01) as well as attention, vigor, and stress scores (all *p* < 0.01) were higher in the afternoon than in the morning. Our results indicated a daily diurnal variation in short-term maximal performance and mood states in young athletic women with better performance observed during the afternoon.

## 1 Introduction

Recent data in chronobiology studies among athletes have confirmed that physiological, psychological, and performance parameters during physical exercise could be time of day (TOD) dependent on a fluctuation in performance from morning to afternoon (Lopes-Silva et al., 2019; Chtourou et al., 2018; Guette et al., 2005; Saidi et al., 2021). The effect of TOD on short-term maximal anaerobic performance has been demonstrated, in men, for various tasks such as the Wingate test (Chtourou et al., 2011; Souissi et al., 2004), the repeated sprint ability test (Chtourou et al., 2011; Aloui et al., 2013), the vertical jump (López-Samanés et al., 2016; Chtourou and Souissi, 2012; Chtourou et al., 2013) or very brief all-out efforts, such as maximal voluntary contraction (Krčmárová et al., 2018; Sedliak et al., 2008). For instance, during the Wingate test, it has been well documented that the peak power (Ppeak) and mean power (Pmean) show TOD fluctuations, with morning nadirs, afternoon/early evening the highest values (Souissi et al., 2004; Souissi et al., 2007; Souissi et al., 2012) and a peak-to-trough amplitude equal to 7.6% ± 0.8% and 11.3% ± 1.1%, respectively (Souissi et al., 2004).

In fact, this greater productivity in the afternoon might be attributed to the time at which the body temperature is at its circadian peak, the circadian phenotype, the internal biological time (the entrained wake-up time), and the fluctuation of cognitive performance (Bougrine et al., 2022). However, this daily fluctuation in performance can be altered by several factors such as the type and intensity of exercise, age, nutritional status, training schedule, level of physical activity, and sleep deprivation (Saidi et al., 2021; Mejri et al., 2015; Souissi et al., 2020). Additionally, the variations in hormonal climate that accompany the different menstrual cycles in women could certainly affect the characteristics of diurnal fluctuations in sports performance (Mejri et al., 2015; Souissi et al., 2020; Carmichael et al., 2021; Julian et al., 2017; Romero-Moraleda et al., 2017; Giacomoni et al., 2000; Sutresna, 2016).

Interestingly, extensive research has been conducted on the circadian or daily fluctuations in the thermoregulatory, cardiovascular, ventilation, metabolic, and hormonal responses of healthy men, both at rest and during exercise. However, there is a notable scarcity of similar studies focusing on women (Tounsi et al., 2018; Graja et al., 2020). In this context, previous studies that were conducted among female athletes demonstrated better performance during afternoon sessions concerning short-term high-intensity performance (Bougrine et al., 2022; Graja et al., 2020; Oleka, 2020). In this context, previous studies revealed that cognitive and high-intensity physical performance among female athletes depend of time of day with improvements observed in the afternoon (Bougrine et al., 2022; Bougrine et al., 2023; Bougrine et al., 2024). Nonetheless, during the past few decades, women’s participation in physical activity and sports has increased substantially. Studies on women’s performance have not kept up with this exponential increase in women engaging in a wide range of physical activities, from recreational sports to extremely strenuous elite sports (“IOC—International Olympic Committee Olympics.com,” 2022). Given the notable anatomical, physiological, and endocrinological differences between male and female athletes, it is crucial to recognize that identical outcomes may not apply to both genders. Providing specific recommendations for each gender would be valuable for both athletes and coaches, enabling a deeper understanding of these daily variations in mood and physical performance and facilitating more informed planning of training and competitions. On the other hand, to the best of the authors’ knowledge, few studies have investigated the effect of time of day among females on cognitive performance (Tounsi et al., 2018; Oleka, 2020).

Additionally, previous studies reported that cognitive and physical activities fluctuated over the day in terms of cognitive abilities, reaction time, strength, and body temperature (Oleka, 2020; Bougrine et al., 2023). Some of these factors can influence dynamic equilibrium and could create daily fluctuations in this aspect of neuromuscular control (Mhenni et al., 2017; Abdessalem et al., 2019). However, little is known about the influence of TOD on motor capacity such as dynamic balance.

The complex interactions between the physiological and psychological components of sports performance have been the subject of extensive recent research. The study conducted by Navabinejad et al. (Navabinejad and Rostami, 2023) delves into the intricate relationship between psychological factors and physical performance. The findings highlight the significant influence of emotions, cognition, and mental strategies on critical parameters such as muscular tension and overall sports performance (Dewi and Makia, 2023). These investigations have demonstrated the significant impact that stress and anxiety have on controlling vital physiological processes such as heart rate, respiration, and muscle contraction in athletes, leading to appreciable declines in performance performance (Uroh and Adewunmi, 2021). In addition, Kadi et al. (Kadi et al., 2023) revealed a significant improvement in dart throwing performance during afternoon sessions compared to morning sessions, along with a decrease in perceived difficulty. Furthermore, a negative correlation was observed between evening dart scores and perceived difficulty scores among those in the evening group, showing that evening performance was perceived as less difficult.

In order to explain the diurnal variation in physical performance and psychological variables, previous studies have examined the effect of TOD on mood states (Chtourou et al., 2018; Souissi et al., 2020; Gribble et al., 2007). Souissi et al. (Souissi et al., 2020) have shown that negative mood states (i.e., tension, depression, anger, fatigue, and confusion) and total mood disturbance (TOD) are higher in the morning compared to the afternoon; however, vigor is greater in the afternoon compared to the morning. Likewise, in elite judokas, Chtourou et al. (Chtourou et al., 2018) showed that vigor was significantly higher in the afternoon compared to the morning. However, this study did not show a significant difference between the two times of day or anxiety, anger, confusion, depression, fatigue, interpersonal relationships, and the Profile of Mood States (POMS) total score. However, prepubescent boys reported no difference between morning and afternoon for vigor and negative moods (Chtourou et al., 2014).

Thus, the aim of the present study was to investigate the effect of TOD on short-term maximal performance, dynamic balance, attention, and psychological variables (i.e., mood) in young women. In light of the available published data, our hypothesis is that TOD may impact the dynamic balance, attention, psychological variables, and short-term high-intensity performances among young females.

## 2 Methods

### 2.1 Participants

Fifteen amateur women actively engaged in various sports disciplines [gymnastics (*n* = 5), judo (*n* = 4), handball (*n* = 3), karate (*n* = 3)] (age: 22 ± 3 years, height:162 cm ± 3.5 cm, weight:58 ± 4 kg, BMI:22, 5 ± 2.2; Leg length:86.73 ± 4.3; 6 ± 2, 18 h of training/week; 8 ± 1, 9 years of training experience; mean ± standard deviation) volunteered to participate in the study. They were fully informed about the study aims and procedures, and they provided written informed consent before testing. Also, they were free to withdraw from the study at any time without further consequences. The study protocol complied with the Code of Ethics for human experimentation of the World Medical Association and the Declaration of Helsinki (World Medical Association, 2013) and the ethical and procedural requirements for Human Chronobiology research (Faul et al., 2007). The experimental protocol was pre-approved by the ethical review board of the Institute.

Following the recommended guidelines proposed by Beck et al. (Beck, 2013), we used G*Power software (version 3.1.9.6; Kiel University, Kiel, Germany) (Faul et al., 2007) to pre-determine the required sample size. The significance level (α) was established at 0.05, with a desired statistical power (β) of 0.95. Based on Bougrine et al. (Bougrine et al., 2023) and discussed between authors, we approximated the effect size to be 0.5. To attain the requisite statistical power, it was determined that a sample size of at least 15 athletes would be sufficient, therefore minimizing the probability of a type 2 statistical error. To mitigate dropout rates, we screened 32 surveys and pinpointed 21 participants deemed appropriate for inclusion in the investigation. Despite this, six participants opted to withdraw from the study. Consequently, fifteen participants met the study criteria and were included in our analysis.

Participants were non-smokers and did not consume caffeine or alcoholic beverages that could improve or impair alertness. It excluded pregnant women, those who had been injured or had surgery in the previous year, those who were severely dizzy or had vestibular problems, and those on balance-affecting medications (psychotropics, hypnotics, or antidepressants). Participants were not using any forms of contraception, including patches, oral medications, injections, implants, or intrauterine devices, and did not have any menstrual or endocrine disorders during the last 3 months prior to the testing sessions. Given that circadian typology could influence the study outcomes, athletes were chosen based on their chronotype using Horne and Östberg’s “morningness/eveningness” questionnaire, employing a 5-category scale (Horne and Östberg, 1977). To ensure homogeneity, all participants exhibited an intermediate circadian typology, with scale scores falling within the range of 43–56.

### 2.2 Experimental design

During the week before the experiment, all subjects came to the laboratory several times at different hours of the day to undergo anthropometric measurements and to become fully familiarized with the procedure and tests involved to minimize potential learning effects during the experiment (Edwards et al., 2013; Pullinger et al., 2018). Subjects were performed in two experimental trials: one in the morning between 07:00–08:30 h and one in the evening between 16:00 and 17:30 h in random order on non-consecutive days (i.e., approximately 48 h separated each test day). These time points, i.e., 07:00–08:30 h and 16:00–17:30 h, were chosen because they are generally reported in the literature as phases of the minimum and maximum daytime levels of power output during a 30-s maximal cycling exercise, respectively (Souissi et al., 2004).

To prevent any masking effect caused by different phases of the menstrual cycle and/or to contrast metabolic responses, both morning and evening tests were conducted at the same menstrual cycle phase (i.e., during the follicular phase between days 6 and 9 of the menstrual cycle) as recommended by Reilly and Bambaeichi et al. (Bambaeichi et al., 2004). The cycles were observed through women’s menstrual diaries, documenting the date and duration of menstruation.

**FIGURE 1:**
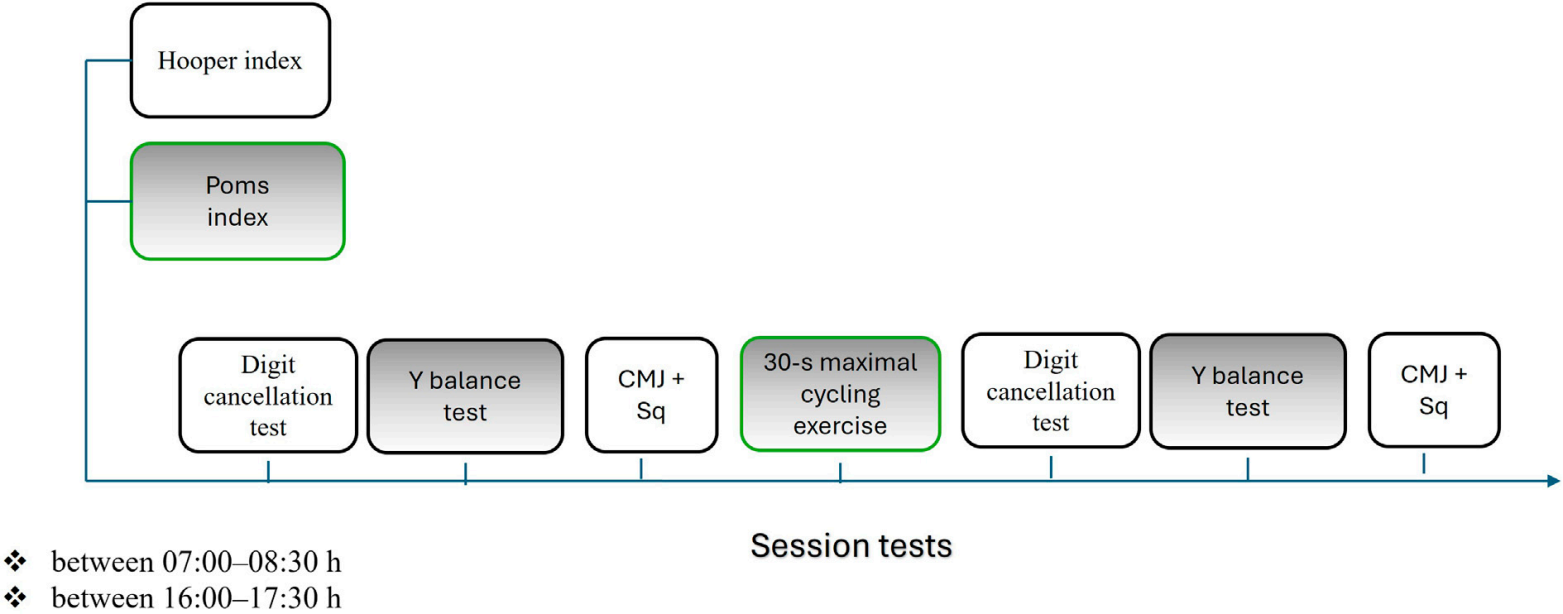
Experimental protocol and timeline of testing procedures. CMJ, countermovement jump; Sq, squat jump.

During each test session, the subjects completed the sleep quality, fatigue, stress, muscle soreness (hooper) and the French version of POMS (POMS-F) questionnaires. After that, they performed a 30-s maximal cycling exercise. The digit cancellation test, SJ and CMJ tests and the lower quarter Y balance test were performed at the start and at the end of each session (Figure 1).

To minimize confounding factors, instructions related to sleep and diet were given to the subjects before the experiment, as recommended by Bougard et al. (Bougard et al., 2009). Throughout the experimental period, participants were requested to maintain their habitual physical activity and to avoid strenuous activity during the 24 h before the test sessions. Subjects were asked to keep their usual sleeping habits, with a minimum of 6 h sleep taken the night preceding each test session. These specific times of the day were selected as they roughly correspond to the batyphase and acrophase, representing peak short-term performance, cognitive function, and oral temperature for this chronotype (neither chronotype) (Bougrine et al., 2022; Bougrine et al., 2023; Chtourou et al., 2014).Before the morning test session, participants were instructed to wake up at 06:00 h. They were fasting and allowed to drink only one glass of water (15–20 cL). Before the evening test sessions, all subjects had the same standard isocaloric meal at 12:00 h, which was concluded at least 5 h before performing the test. During the period of the investigation, the subjects were not allowed to consume food, beverages, or any known stimuli (e.g., caffeine) or depressants (e.g., alcohol) that could possibly enhance or compromise alertness.

#### 2.2.1 30-s maximal cycling exercise

This test consists of a pedaling exercise on an ergometric bicycle (Monark 894e Stockholm, Sweden) at maximum speed for 30 s against a constant braking force established as a function of body weight using optimization tables from Bar-Or et al. (Bar‐Or, 1987) (0.084 kg per kilogram of body weight). Each subject is securely attached at the level of the pedals with toe clips and straps to prevent their feet from slipping off the pedal. Strong verbal motivation was given to participants during the test. The height of the saddle was adjusted to the satisfaction of each participant. This height was recorded and used for each participant in all tests.

The test allows us to identify three parameters:

Peak power, which is the highest mechanical power typically seen in the first 5–6 s. The power is calculated as follows: *p* (Watt) = F (kg) × V (t.min −1), where *p* is the peak or mean power in Watt; F is the braking force in kilograms; V is the pedaling speed in revolutions per minute. The mean power developed during 30 s of the test. The Fatigue Index (FI) is the ability to maintain a high percentage of peak power for 30 s. It is calculated using the Bar-Or formula in 1987: Fatigue index (%) = [(peak power—minimal power)/peak power) × 100.

#### 2.2.2 Squat jump (SJ) and countermovement jump (CMJ) tests

Jumping performances were assessed with an infrared jump system (Optojump, Microgate, Bolzano, Italy) interfaced with a microcomputer. The Optojump system measures flight and contact times with an accuracy of 1/1,000 of a second. For the SJ tests, the subjects started in the semi-squat position with a knee flexion angle of nearly 90° and without moving, and the subjects had their hands placed on their hips. At the signal, the subjects pushed off. CMJ tests started in an upright standing position with the subjects’ hands on their hips. At the signal, the subjects made a downward movement until reaching an approximate knee angle of 90°, and subsequently began to push-off. Each subject performed three jumps in SJ and CMJ with 3 minutes of rest in between to avoid any fatigue effect, and the best jump was selected for analysis. Every subject was given a 15-s interval between attempts.

#### 2.2.3 Y balance test

The lower quarter Y balance test (YBT) was used to measure dynamic balance, as previously described by Robinson et al. (Robinson and Gribble, 2008). And Blasco et al. (Blasco et al., 2019). It was developed to refine the Star Excursion Balance Test (SEBT). The YBT uses the anterior (A), posteromedial (PM), and posterolateral (PL) components of the SEBT to provide an accurate assessment of lower limb neuromuscular control such as coordination, balance, flexibility, and strength. The posterior directions are each positioned 135° from the anterior direction and 90° between them.

Since shoe variations can make it difficult to standardize test results, it is recommended that the test be performed barefoot. The YBT requires the athlete to place their hands on their hips and to balance on one leg whilst simultaneously reaching as far as possible with the other leg in three directions. The distance from the center footrest to the tip of the stretched leg was measured in centimeter units. Three trials were performed in each direction, and the longest distance for each direction was used for analysis. The YBT score was calculated by summing the maximal reach for each direction and leg and normalizing the results to limb length (Blasco et al., 2019). Limb length was measured in the supine position, from the anterior-superior iliac spine to the center of the medial malleolus (Gribble et al., 2007). The following formula was used:

YBT = (maximum anterior reach distance + maximum posteromedial.

Reach distance + maximum posterolateral reach distance)/(leg length × 3) × 100 (%)

#### 2.2.4 Profile of mood states (POMS)

The French version of the self-reported POMS questionnaire was used to evaluate subjective mood states. It consists of 65 adjectives developed to measure seven aspects of mood (i.e., tension, depression, anger, vigor, fatigue, confusion, and interpersonal relationships) from 65 adjectives (McNair et al., 1971). Responses to each item ranged from “0” (not at all) to “4” (extremely), with higher scores indicating a more negative mood state.

#### 2.2.5 The Hooper questionnaire

Before each session, participants were asked to give information about their subjective assessment of the sleep quality of the previous night, as well as ratings of fatigue, stress, and muscle soreness. This strategy allows the assessment of the wellness state of the participants (Hooper et al., 1995). The four subjective ratings were recorded on a Likert scale ranging from one to seven and from “very, very good” to “very, very bad” for sleep, and from “very, very low” to “very, very high” for fatigue, stress, and muscle soreness. The Hooper Index reflects the sum of the four ratings.

#### 2.2.6 The digit cancellation test

The digit cancellation test provides a highly practical and user-friendly assessment of various aspects of prefrontal cortical functioning, including processing speed of information, attention focus and executive function. The test is composed of four pages (Hatta et al., 2012). Each page contains 600 numbers of one–five digits arranged in 36 lines. Participants performed the digit-cancellation test for 1 minute, deleting target numbers (i.e., numbers composed of three grouped digits) on a sheet of randomly arranged possibilities. The sum of the correctly deleted numbers was registered for analysis.

### 2.3 Statistical analysis

All statistics were analyzed using Statistics for Windows version 10. The values are expressed as the mean ± standard deviation (M ± SD). The normality of the distributions was tested with the Shapiro-Wilk test. Once the normality assumption was confirmed (*p* > 0.05), parametric tests were performed. A paired Student’s t-test was used to examine the differences between morning and evening for the Wingate test (Ppeak, Pmean, and FI), the data from the Hooper and the POMS-f questionnaires. CMJ, SJ, the digit cancellation test, anterior reach distance, posteromedial reach distance, posterolateral reach distance, and the YBT score were analyzed using a two-way analysis of variance (ANOVA) (TOD effect × exercise) with repeated measures. For each of the analyses, when the ANOVA showed a significant effect, a Bonferroni post-hoc test was applied to compare the experimental data in pairs. Statistical significance was established at *p* < 0.05.

## 3 Results

### 3.1 30-s maximal cycling exercise

Statistics analysis showed a significant TOD effect on the Ppeak (*t* = 3.68, *p* < 0.001) and Pmean (*t* = 2.47, *p* < 0.01) during the Wingate test were significantly lower in the morning than in the afternoon (*p* < 0.05), while for FI, no significant effect of TOD (*t* = 0.46, *p* > 0.05) was reported (Table 1).

**TABLE 1:** Means ± SD values of peak power (Ppeak), mean power (Pmean) and fatigue index (FI) observed during morning and afternoon (*n* = 15).

	Morning	Afternoon	*p*
Ppeak (W∙kg-1)	103.2 ± 6.74	113.6 ± 8.78	0.001 **^a^**
Pmean (W∙kg-1)	74.88 ± 7.52	80.25 ± 8.7	0.01**
FI (%)	58.86 ± 6.4	60.32 ± 13.3	0.9

^a^ (*p* < 0, 001); **(*p* < 0, 01): significant difference compared to the morning.

### 3.2 Vertical jumps

#### 3.2.1 Countermovement jump

ANOVA revealed a significant TOD effect [F (1.14) = 13.41; *p* < 0.01, ɳp2 = 0.48] on height performance during CMJ. Likewise, there is a significant effect of exercise [F (1.14) = 13.32; *p* < 0.01 ɳp2 = 0.48]. However, the interaction between TOD × exercise was not significant [F (1.14) = 0.41; *p* = 0.53, ɳp2 = 0.02]. The *post hoc* LSD test showed that the height of jump during CMJ was significantly higher in the afternoon than in the morning before (*p* < 0.01) and after the Wingate test (*p* < 0.001) (Table 2).

**TABLE 2:** Means ± SD values of CMJ, SJ before and after exercise observed during morning and afternoon (*n* = 15).

Variable	Morning		Afternoon	
	Before	After	Before	After
CMJ (cm)	23.9±2.62	25.56±2.97	25.84±3^**^	27.07±2.19^&&^
SJ (cm)	23.55±3.04	25.02±2.93	25.35±2.41***	26.66±1.97^&&&^

*** (*p* < 0.001); **(*p* < 0.01): significant difference compared to the morning before the exercise;

^&&&^ (*p* < 0.001);

^&&^ (*p* < 0.01): significant difference compared to the morning after the exercise; cm, centimeter.

#### 3.2.2 Squat jump

ANOVA showed a significant effect of TOD [F (1.14) = 13.21; *p* < 0.01, ɳp2 = 0.48] and of exercise [F (1.14) = 18.67; *p* < 0.001, ɳp2 = 0.57]. However, the interaction of TOD × exercise was not significant [F (1.14) = 0.11; *p* = 0.74, ɳp2 = 0.007]. The *post hoc* LSD test demonstrated that the performance of the SJ was significantly higher in the afternoon than in the morning before (*p* < 0.001) and after (*p* < 0.001) the Wingate test (Table 2).

### 3.3 Y balance test

For the AT reach direction, there was no TOD × exercise interaction [DL: F (1.14) = 0.32; *p* = 0.57, ɳp2 = 0.02; NDL: F (1.14) = 0.46; *p* = 0.505, ɳp2 = 0.03, respectively]. Nonetheless, a main effect of TOD was found [DL: F (1.14) = 37.57; *p* < 0.001, ɳp2 = 0.72; NDL: F (1.14) = 12.55; *p* < 0.01, ɳp2 = 0.47, respectively]. Likewise, there is a significant exercise effect (DL: F (1.14) = 16.31; *p* < 0.01, ɳp2 = 0.53; NDL: F (1.14) = 11.25; *p* < 0.01, ɳp2 = 0.44, respectively). The post-hoc test revealed that the AT scores “before exercise in the afternoon” were better (*p* < 0.05) than “before exercise in the morning” for DL, however no difference in the AT scores for NDL (*p* > 0.05) (Table 3).

**TABLE 3:** Means ± SD values of composite score (Cs) anterior (AT), posterolateral (PL) and posteromedial (PM) for the dominant (DL) and non-dominant legs (NDL) during the balance test before and after the exercise observed during morning and afternoon (*n* = 15).

Variable	Morning		Afternoon	
	Before	After	Before	After
AT DL (cm)	70.13 ± 4.01	73.2 ± 3.00	73.8 ± 2.98*	76 ± 4.65
A^a^ NDL (cm)	69.66 ± 5.24	72.73 ± 4.77	72.93 ± 5.07	74.93 ± 3.05
PM DL (cm)	73.4 ± 5.08	76.53 ± 4.37	77.47 ± 4.48**	81 ± 4.15^&&^
PM NDL (cm)	75.53 ± 5.55	78.13 ± 4.05	78.2 ± 5.15*	79.93 ± 4.93
PL DL (cm^a^	72.86 ± 4.3	76.06 ± 3.3	76.46 ± 3.71**	79.46 ± 4.73^&&^
PL NDL (cm)	75.13 ± 3.7	77.46 ± 3.44	77.4 ± 5.01*	78.6 ± 5.12
CS DL (%LL)	83.33 ± 4.7	^a^6.33 ± 3.35	86, 33 ± 3.58*	90.39 ± 4.02^&&^
CS NDL (%LL)	84.79^a^4.48	87.9 ± 5.07	87.94 ± 5.21**	89.84 ± 4.14^&&&^

^*^ (*p* < 0.05); **(*p* < 0.01): significant difference for comparison of the morning before the exercise.

^&&^ (*p* < 0.01).^&&&^(*p* < 0.001): significant difference for comparison of the morning after the exercise.

For the PM reach direction, there was no TOD × exercise interaction [DL F: (1.14) = 0.08; *p* = 0.77, ɳp2 = 0.006; NDL: F (1.14) = 0.51; *p* = 0.48, ɳp2 = 0.03, respectively]. Nonetheless, a main effect of TOD was found [DL: F (1.14) = 21.36; *p* < 0.001, ɳp2 = 0.60]; NDL: F (1.14) = 8.21; *p* < 0.01, ɳp2 = 0.36, respectively). Likewise, there is a significant exercise effect {DL: [F (1.14) = 15.21; *p* < 0.001, ɳp2 = 0.52]; NDL: F (1.14) = 7.77; *p* < 0.05, ɳp2 = 0.35, respectively}. The post-hoc test revealed that the PM scores “before exercise in the afternoon” were better (DL: *p* < 0.01; NDL: *p* < 0.05) than the “before exercise in the morning” for both legs. Additionally, the test showed that PM scores “After exercise in the afternoon” was better for DL: *p* < 0.001 than the “after exercise in the morning”, however no difference in the AT scores for NDL (*p* > 0.05) (Table 3).

For the PL reach direction, there was no TOD × exercise interaction [DL: F (1.14) = 0.03; *p* = 0.86, ɳp2 = 0.002; NDL: F (1.14) = 1.22; *p* = 0.28, ɳp2 = 0.08, respectively]. Nonetheless, a main effect of TOD was found [DL: F (1.14) = 13.26; *p* < 0.01, ɳp2 = 0.48; NDL: F (1.14) = 6.23; *p* < 0.05, ɳp2 = 0.30, respectively]. Likewise, there is a significant exercise effect [DL: F (1.14) = 16.46; *p* < 0.01, ɳp2 = 0.54; NDL: F (1.14) = 5.28; *p* < 0.05, ɳp2 = 0.27, respectively]. The post-hoc test revealed that the PL scores “before exercise in the afternoon” were better (DL: *p* < 0.01; NDL: *p* < 0.05) than “before exercise in the morning” for both legs. Additionally, the test showed that PL scores “After exercise in the afternoon” was better for DL: *p* < 0.01 than the “after exercise in the morning” for both legs, however no difference in the AT scores for NDL (*p* > 0.05) (Table 3).

For CS there was no TOD × exercise interaction [DL: F (1.14) = 0.67; *p* = 0.42, ɳp2 = 0.04; NDL: F (1.14) = 2.1; *p* = 0.16, ɳp2 = 0.13, respectively]. Nonetheless, a main effect of TOD was found [DL: F (1.14) = 59.62; *p* < 0.001, ɳp2 = 0.8; NDL: F (1.14) = 16.99; *p* < 0.01, ɳp2 = 0.54, respectively]. Likewise, there is a significant effect of these test variables during exercise [DL: F (1.14) = 17.25; *p* < 0.001, ɳp2 = 0.55; NDL: F (1.14) = 25.72; *p* < 0.001, ɳp2 = 0.64, respectively]. The post-hoc test revealed that the CS scores “before exercise in the afternoon” were better (DL: *p* < 0.05; NDL: *p* < 0.001) than the “before exercise in the morning” for both legs. Additionally, the test showed that CS scores “After exercise in the afternoon” was better (DL: *p* < 0.01; NDL: *p* < 0.001) than the “after exercise in the morning” for both legs (Table 3).

### 3.4 Profile of Mood States questionnaire

Statistics analysis showed a significant TOD effect (*t* = 4.29, *p* < 0.001) on the vigor scores which were significantly higher in the afternoon than in the morning (*p* < 0.001). However, no significant difference in times of day was recorded for anxiety (*t* = 2.12, *p* > 0.05), anger (*t* = 1.67, *p* > 0.05), confusion (*t* = 1.65, *p* > 0.05), depression (*t* = 1.38, *p* > 0.05), fatigue (*t* = 0.29, *p* > 0.05), interpersonal relationships (*t* = 1.84, *p* > 0.05), and TMD (*t* = 1.90, *p* > 0.05) (Table 4).

**TABLE 4:** Means ± SD values of Anxiety, anger, confusion, depression, fatigue, vigor, interpersonal relationship, and total mood disturbances (TMD) scores registered by the Profile of Mood State questionnaire recorded in the morning and the afternoon (*n* = 15).

	Morning	Afternoon	*p*-value
Anxiety (a.u.)	8, 93 ± 8, 01	11 ± 6.35	0.06
Anger (a.u.)	14.06 ± 9.8	15, 86 ± 11.21	0.1
Confusion (a.u.)	10, 66 ± 5.58	11, 6 ± 5.48	0.1
Depression (a.u.)	11.73 ± 6.4	14.06 ± 11.34	0.1
Fatigue (a.u.)	11, 66 ± 6.21	10, 93 ± 4.49	0.7
Vigor (a.u.)	12, 13 ± 5.13	17.26 ± 5.2	0.001***
Interpersonal relationship (a.u.)	15.66 ± ^a^	15.93 ± 4.99	0.08
Emotional distress scores (a.u.)	40.2 ± 27.77	47.53 ± 35.41	0.07

^***^ (*p* < 0, 001): significant difference compared to the morning; a. u., arbitrary unit.

### 3.5 Hooper questionnaire

Statistics analysis showed a significant TOD effect on Stress scores (*t* = 2.58, *p* < 0.05), with significantly higher in the afternoon than in the morning (*p* < 0.01). However, no significant difference between the time of the day was observed for fatigue (*t* = 1, *p* > 0.05), sleep (t = 1.04, *p* > 0.05), muscle soreness (*t* = 0.56, *p* > 0.05), and the hooper index (*t* = 0.33, *p* > 0.05) (Table 5).

**TABLE 5:** Means ± SD values of Fatigue, stress, sleep, and muscle soreness registered by the Hooper questionnaire recorded in the morning and the afternoon (*n* = 15).

	Morning	Afternoon	*p*-value
Fatigue (a.u.)	4.46 ± 1.24	4.2 ± 1.08	0.4
Stress (a.u.)	4.73 ± 0.96	4.2 ± 1.5	0.02**
Sleep (a.u.)	3.86 ± 1.3	4.6 ± 0.98	0.3
Muscle soreness (a.u.)	3.93 ± 1.33	4.13 ± 1.26	0.58
Total (a.u.)	17 ± 3.54	17.33 ± 2.84	0.7

^**^ (*p* < 0.01): significant difference compared to the morning; a. u.: arbitrary unit.

### 3.6 The digit cancellation test

**FIGURE 2:**
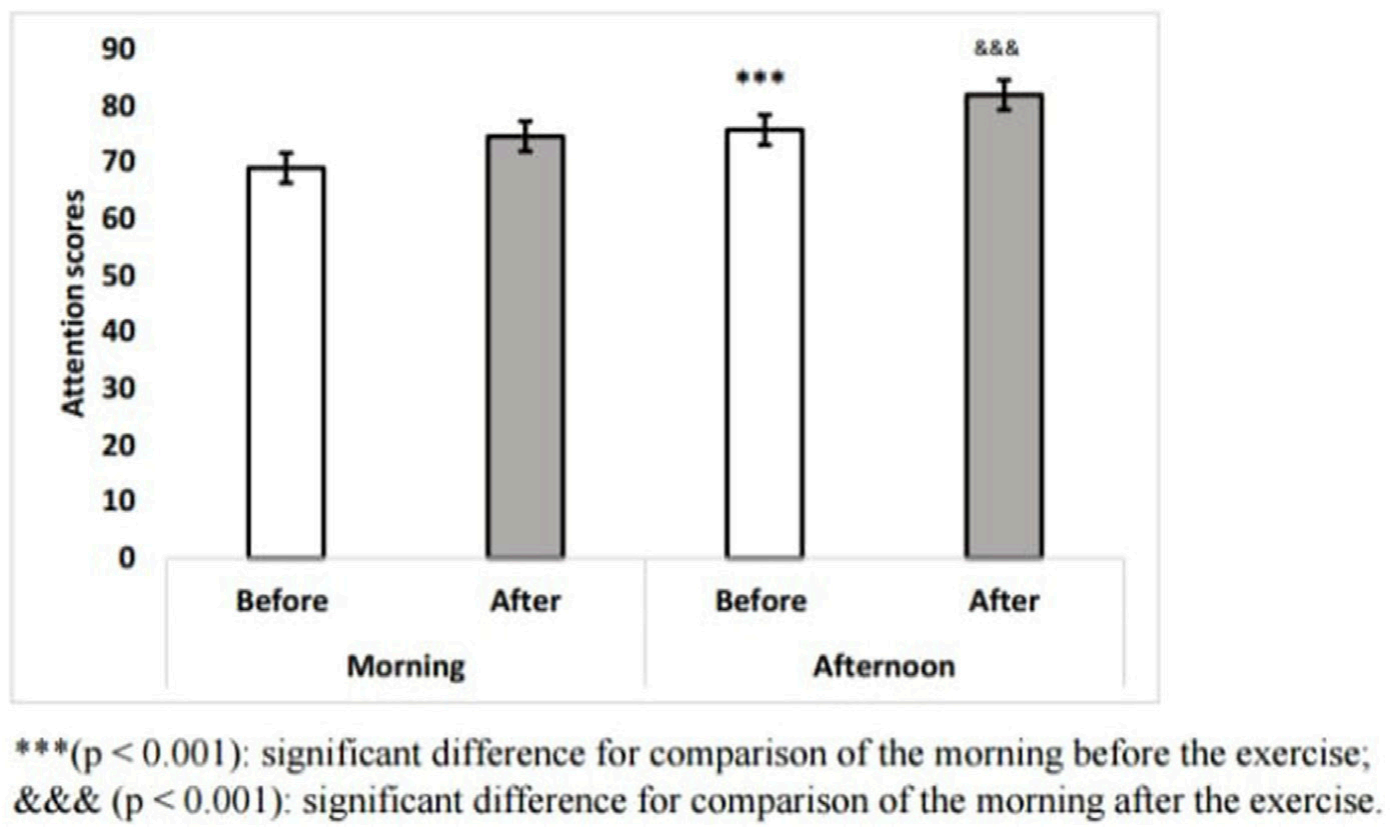
Means ± SD scores of attention test before and after exercise during morning and afternoon. ***(*p* < 0.001): significant difference for comparison of the morning before the exercise; andandand (*p* < 0.001): significant difference for comparison of the morning after the exercise (*n* = 15).

Statistics analysis showed a significant TOD [F (1.14) = 16.36; *p* < 0.01, ɳp2 = 0.53], and exercise effect [F (1.14) = 21.33; *p* < 0.001, ɳp2 = 0.6] on attention. However, no significant interaction [F (1.14) = 0.04; *p* = 0.84, ɳp2 = 0.001] between TOD × exercise was reported. (Figure 2). The *post hoc* LSD test showed that attention was significantly higher in the afternoon than in the morning before (*p* < 0.001) and after the Wingate test (*p* < 0.05) (Figure 2).

## 4 Discussion

The present study investigated diurnal variation in short-term maximal performance, dynamic balance, attention, and psychological variables in females. The main data of our study indicated no morning-afternoon differences in fatigue index, negative mood states as well as fatigue, sleep, and muscle soreness. However, attention, vigor and stress were highest in the afternoon. Likewise, Ppeak and Pmean during the Wingate test and the composite score during the Y balance test were higher in the afternoon compared to the morning.

Regarding cognitive function, the results of the present study agree with those of Valdez et al. (Valdéz et al., 2012) who reported that attention fluctuates throughout the day with the highest attention values from 10:00 h to 14:00 h and from 16:00 h to 22:00 h. In the same context, previous studies have shown that cognitive performance is weak early in the morning and then increases in the afternoon among male (Edwards et al., 2013; Pullinger et al., 2018) and female athletes (Bougrine et al., 2023). Several studies have shown that men’s performance during short-term high-intensity exercise depends on the TOD, peaking around 16:00 h to 20:00 h (Souissi et al., 2012; Ayala et al., 2021). Regarding female athletes, recent studies indicated a better performance in short term high intensity performance during afternoon sessions compared to morning sessions (Bougrine et al., 2022; Bougrine et al., 2023; Mhenni et al., 2017).

Moreover, as part of an experiment that involved five repetitions of maximum pedaling for 6 seconds (with a recovery period of 24 s), Chtourou et al. (Chtourou and Souissi, 2012) found that the maximum power was significantly higher in the afternoon (17:00 h) than in the morning (07:00 h) during only the first two sprints. Furthermore, Souissi et al. (Souissi et al., 2007) reported a higher Ppeak and Pmean in the afternoon than in the morning during the Wingate test. Similarly, Lericollais et al. (Lericollais et al., 2021) and Souissi et al. (Souissi et al., 2004) reported that Ppeak and Pmean in the Wingate test fluctuated with TOD, with acrophases observed at 17:24 h and (18:00 h) and amplitudes of 7.6% ± 0.8% and 11.3% ± 1.1%, respectively. They also reported that the FI was higher in the afternoon compared to the morning (Melhim, 1993; Facer-Childs and Brandstaetter, 2015). In the same context, the vertical jump during CMJ, and SJ test fluctuates with the TOD with values higher in the afternoon than in the morning (Bougrine et al., 2022; Bougrine et al., 2023; Bambaeichi et al., 2004; Bougard et al., 2009). A higher jump was found to occur during the squat jump and countermovement in the afternoon (between 16:00 h and 20:00 h) than in the morning (between 18:00 h and 22:00 h) (Souissi et al., 2004; Mejri et al., 2015; Abdessalem et al., 2019). Our results contrast with the results of Chtourou et al. (Chtourou et al., 2018) which showed that short-term performance, among men, during repeated sprint performances was not different between morning and afternoon. The discrepancies between the present study’s findings and those of Chtourou et al. (Chtourou et al., 2018) could be related to the differences in the involved muscle groups (i.e., cycling in the present study vs sprint running and in the previous studies) and the sex difference could cause this difference (i.e., females in the present study vs males in the previous studies).

Additionally, women’s performance during repeated sprint ability (RSSA) and the five-jump test was significantly influenced by TOD, according to Tounsi et al. (Tounsi et al., 2018) compared to the morning, women soccer players had shorter RSSAs in the afternoon. While the performance of the 5JT was significantly higher in the afternoon compared to the morning. Melhem et al. (Melhim, 1993) investigated the peak and mean power of female physical education students during a Wingate test conducted at four different times as follows: 03:00, 09:00, 15:00, and 21:00. Performance varies throughout the day, as reported by these authors. Peak power at 15:00 was around 7% higher than at 03:00, and mean power at 15:00 and 21:00 was about 16% and 15% higher than at 03:00. However, Bambaeichi et al. (Bambaeichi et al., 2004) found that peak torque values for knee isometric extensors did not vary significantly depending on the TOD under voluntary conditions in sedentary females. In this study, subjects had a higher level of physical activity than in Bambaeichi’s study, which may explain the discrepancy. The training of muscles could increase the muscle’s temporal responses in accordance with TOD by increasing the amount of fast-twitch fibers in the muscles.

Indeed, the increased productivity in the afternoon could be ascribed to the period when the body temperature reaches its circadian peak, the circadian phenotype, and the internally synchronized wake-up time (Chtourou et al., 2013; Bougrine et al., 2022; Bougrine et al., 2023; Facer-Childs and Brandstaetter, 2015). Moreover, these better physical performances in the afternoon compared to the morning can be partly explained by the vigor scores observed by the POMS-F questionnaire, which were better in the afternoon (*t* = 4.29, *p* < 0.001) than in the morning. Furthermore, the present study did not report a significant difference between morning and afternoon for negative mood (*p* > 0.05), fatigue (*p* > 0.05), sleep (*p* > 0.05), and muscle pain (*p* > 0.05) estimated by the Hooper questionnaire.

In our study, we found that the lowest scores for the Y balance test were observed in the morning and the highest scores were obtained in the afternoon. These results are in agreement with those of Heinbaugh et al. (Heinbaugh et al., 2015) which showed that the dynamic balance recorded during the Y balance test fluctuates during the day with a better performance recorded in the afternoon in recreational athletes. Contrary to the results of these studies, Gribble et al. (Gribble et al., 2007) applied a Dynamic Balance Test (SEBT) to 30 college-age participants. They reported that dynamic balance abilities changed at different times of the day, with the best performance in the morning. Likewise, Kwon et al. (Kwon et al., 2014) showed that the TOD had an influence on dynamic postural balance, the performance of this task being better in the morning.

The difference between the results of this study and those of Gribble and al. (Gribble et al., 2007). and Kwon and al. (Kwon et al., 2014) could be related to the participating population. Previous studies have suggested that regular training at a specific TOD can increase performance at that specific TOD (Chtourou and Souissi, 2012). Midway through our data collection, all participants informed us that they were playing sports in the afternoon, so the peak in balance performance observed in the afternoon could be due to the acclimatization of their body to exercise at this TOD.

Like any scientific investigation, the current results come with certain limitations. This study specifically concentrated on “short-term” physical performance. Therefore, future empirical studies should focus on the aerobic performance of female athletes, perhaps by evaluating parameters like VO2max, to gain insights into how a woman’s body responds to various exercises. Given that our study focuses on assessing short-term maximal performance via cycling and jump tests, incorporating a diverse range of exercise types in future studies could unveil whether time-of-day effects persist consistently across various physical activities. Further, the sample size is relatively small, and future research should test the robustness of findings using larger samples. Additionally, caution should be taken when generalizing our conclusions since the sample used in this study included practitioners of gym, who are not world-ranked athletes. So, it could be recommended for future studies to investigate the effects of the combined interaction between the different phases of the menstrual cycle and the TOD on the physical performance of women. The next experiments must explain these observations. Plasma doses of hormones, EMG recordings, and electrical stimulation of the cortex can be considered to understand the peripheral and central origin of our observations. Similarly, the measurement of muscle temperature can inform us about the links of causality between diurnal fluctuations in temperature and muscle performance. Moreover, since the present study focused exclusively on young, physically active women, its findings cannot be generalized to other demographic groups, such as men, sedentary individuals, or older adults. Future studies should incorporate a wider demographic spectrum, including both male and older participants, to enhance the breadth of understanding regarding diurnal fluctuations in performance and mood. This approach would yield a more comprehensive insight into diurnal physical performance and mood variations. Furthermore, our study does not take into account certain external factors such as sleep quality, menstrual cycle phases, and environmental conditions, which can influence physical and psychological performance. Thus, incorporating more psychological assessments or physiological measures of stress and fatigue in future research could potentially provide deeper insights into the participants’ psychological and physiological states. Also, incorporating more testing times in future studies can enhance our understanding of diurnal variations and performance fluctuations throughout the day Also, incorporating more testing times in future studies can enhance our understanding of diurnal variations and performance fluctuations throughout the day.

## 5 Conclusion

In conclusion, the present study indicated that TOD has significant effects on short-term performance, attention, and psychological variables. Notably, the observed performances demonstrated superior values during the afternoon compared to the morning. Athletes and coaches may benefit from these results in improving training schedule planning and organization. Acknowledging and incorporating the influence of TOD on various performance aspects could helps athletes and coaches to optimize training schedules for improved outcomes. Recognizing that optimal physical and cognitive abilities are times of the day dependent with better improvement during the afternoon informs decisions regarding training and competition timing. It is possible for coaches to strategically plan workouts or skill-based exercises during peak performance periods, maximizing the potential of athletes. Additionally, athletes can take advantage of these outcomes by aligning their training schedules with their circadian rhythms.

## Data Availability

The raw data supporting the conclusion of this article will be made available by the authors, without undue reservation.

## References

[B1] Abdessalem R. Boukhris O. Hsouna H. Trabelsi K. Ammar A. Taheri M. (2019). Effect of napping opportunity at different times of day on vigilance and shuttle run performance. Chronobiol. Int. 36 (10), 1334–1342. 10.1080/07420528.2019.1642908 31368367

[B2] Aloui A. Chtourou H. Masmoudi L. Chaouachi A. Chamari K. Souissi N. (2013). Effects of Ramadan fasting on male judokas’ performances in specific and non-specific judo tasks. Biol. Rhythm Res. 44 (4), 645–654. 10.1080/09291016.2012.722454

[B3] Ayala V. Martínez-Bebia M. Latorre J. A. Gimenez-Blasi N. Jimenez-Casquet M. J. Conde-Pipo J. (2021). Influence of circadian rhythms on sports performance. Chronobiol Int. 38 (11), 1522–1536. 10.1080/07420528.2021.1933003 34060402

[B4] Bambaeichi E. Reilly T. Cable N. T. Giacomoni M. (2004). The isolated and combined effects of menstrual cycle phase and Time-of-Day on muscle strength of eumenorrheic females. Chronobiol. Int. 21 (4-5), 645–660. 10.1081/CBI-120039206 15470960

[B5] Bar-Or O. (1987). The Wingate anaerobic test. An update on methodology, reliability and validity. Sport. Med. Int. J. Appl. Med. Sci. Sport Exerc. 4, 381–394. 10.2165/00007256-198704060-00001 3324256

[B6] Beck T. W. (2013). The importance of *a priori* sample size estimation in strength and conditioning research. J. Strength Cond. Res. 27 (8), 2323–2337. 10.1519/JSC.0b013e318278eea0 23880657

[B7] Blasco J. M. Tolsada C. Beltrán M. Momparler A. M. Sanchiz-Benavente R. Hernández-Guillen D. (2019). Instability training, assessing the impact of level of difficulty on balance: a randomized clinical trial. Gait Posture 70, 116–121. 10.1016/j.gaitpost.2019.02.029 30849606

[B8] Bougard C. Bessot N. Moussay S. Sesbouee B. Gauthier A. (2009). Effects of waking time and breakfast intake prior to evaluation of physical performance in the early morning. Chronobiol Int. 26 (2), 307–323. 10.1080/07420520902774532 19212843

[B9] Bougrine H. Ammar A. Trabelsi K. Belgacem A. Salem A. Chtourou H. (2024). The effect of last meal" suhoor" timing on diurnal variations in cognitive performance during ramadan fasting among female athletes. Front. Nutr. 11, 1373799. 10.3389/FNUT.2024.1373799 38694225 PMC11061406

[B10] Bougrine H. Cherif M. Chtourou H. Souissi N. (2022). Can caffeine supplementation reverse the impact of time of day on cognitive and short-term high intensity performances in young female handball players. Chronobiol. Int. 39 (8), 1144–1155. 10.1080/07420528.2022.2077747 35603451

[B11] Bougrine H. Cherif M. Chtourou H. Souissi N. (2023). Does Ramadan intermittent fasting affect the intraday variations of cognitive and high-intensity short-term maximal performances in young female handball PLAYERS? Biol. Rhythm Res. 54 (4), 399–418. 10.1080/09291016.2023.2198794

[B12] Carmichael M. A. Thomson R. L. Moran L. J. Wycherley T. P. (2021). The impact of menstrual cycle phase on athletes performance: a narrative review. Int. J. Environ. Res. Public Health 18 (4), 1667. 10.3390/IJERPH18041667 33572406 PMC7916245

[B13] Chtourou H. Aloui A. Hammouda O. Chaouachi A. Chamari K. Souissi N. (2013). Effect of static and dynamic stretching on the diurnal variations of jump performance in soccer players. PLoS One 8 (8), e70534. 10.1371/journal.pone.0070534 23940589 PMC3734300

[B14] Chtourou H. Aloui A. Hammouda O. Souissi N. Chaouachi A. (2014). Diurnal variation in long and short-duration exercise performance and mood states in boys. Sport Sci. HLTH 10 (3), 183–187. 10.1007/s11332-014-0190-0

[B15] Chtourou H. Engel F. A. Fakhfakh H. Fakhfakh H. Hammouda O. Ammar A. (2018). Diurnal variation of short-term repetitive maximal performance and psychological variables in elite judo athletes. Front. Physiol. 9, 1499. 10.3389/fphys.2018.01499 30416454 PMC6212582

[B16] Chtourou H. Hammouda O. Souissi H. Chamari K. Chaouachi A. Souissi N. (2011). The effect of Ramadan fasting on physical performances, mood state and perceived exertion in young footballers. Asian J. Sports Med. 2 (3), 177–185. 10.5812/asjsm.34757 22375237 PMC3289213

[B17] Chtourou H. Souissi N. (2012). The effect of training at a specific time of day: a review. J. Strength Cond. Res. 26 (7), 1984–2005. 10.1519/JSC.0b013e31825770a7 22531613

[B18] Dewi I. C. Makia K. R. (2023). The impact of psychological perspective on pandemic for the development of social life. Int. J. Soc. Sci. 6. 10.47191/IJSSHR/V6-I10-38

[B19] Edwards B. J. Pullinger S. A. Kerry J. W. Robinson W. R. Reilly T. P. Robertson C. M. (2013). Does raising morning rectal temperature to evening levels offset the diurnal variation in muscle force production? Chronobiol Int. 30 (4), 486–501. 10.3109/07420528.2012.741174 23281719

[B20] Facer-Childs E. Brandstaetter R. (2015). The impact of circadian phenotype and time since awakening on diurnal performance in athletes. Curr. Biol. 25 (4), 518–522. 10.1016/J.CUB.2014.12.036 25639241

[B21] Faul F. Erdfelder E. Lang A. G. Buchner A. (2007). G* Power 3: a flexible statistical power analysis program for the social, behavioral, and biomedical sciences. Behav. Res. methods 39 (2), 175–191. 10.3758/bf03193146 17695343

[B22] Giacomoni M. Bernard T. Gavarry O. Altare S. Falgairette G. (2000). Influence of the menstrual cycle phase and menstrual symptoms on maximal anaerobic performance. Med. Sci. Sports Exerc 32 (2), 486–492. 10.1097/00005768-200002000-00034 10694136

[B23] Graja A. Kacem M. Hammouda O. Borji R. Bouzid M. Souissi N. (2020). Physical, biochemical, and neuromuscular responses to repeated sprint exercise in eumenorrheic female handball players: effect of menstrual cycle phases. J. strength Cond. Res. 36 (8), 2268–2276. 10.1519/jsc.0000000000003556 32168179

[B24] Gribble P. A. Tucker W. S. White P. A. (2007). Time-of-day influences on static and dynamic postural control. J. Athl. Train. 42 (1), 35–41. 17597941 PMC1896064

[B25] Guette M. Gondin J. Martin A. (2005). Morning to evening changes in the electrical and mechanical properties of human soleus motor units activated by H Reflex and M wave. Eur. J. Appl. Physiol. 95 (4), 377–381. 10.1007/s00421-005-0023-6 16151836

[B26] Hatta T. Yoshizaki K. Ito Y. Mase M. Kabasawa H. (2012). Reliability and validity of the digit cancellation test, a brief screen of attention. Psychologia 55 (4), 246–256. 10.2117/PSYSOC.2012.246

[B27] Heinbaugh E. M. Smith D. T. Zhu Q. Wilson M. Dai B. (2015). The effect of time-of-day on static and dynamic balance in recreational athletes. Sport. Biomech. 14 (3), 361–373. 10.1080/14763141.2015.1084036 26517605

[B28] Hooper S. MacKinnon L. Wilson B. (1995). Biomechanical responses of elite swimmers to staleness and recovery. Aust. J. Sci. Med. Sport 27 (1), 9–13. 7780775

[B29] Horne J. A. Östberg O. (1977). Individual differences in human circadian rhythms. Biol. Psychol. 5 (3), 179–190. 10.1016/0301-0511(77)90001-1 922076

[B30] Julian R. Hecksteden A. Fullagar H. Meyer T. (2017). The effects of menstrual cycle phase on physical performance in female soccer players. PLoS One 12 (3), e0173951. 10.1371/JOURNAL.PONE.0173951 28288203 PMC5348024

[B31] Kadi M. N. Orhan O. Yüksel Y. (2023). Do different times of the day affect dart throwing performance? Int. J. Sport Stud. Health. 6 (2). 10.5812/INTJSSH-142021

[B32] Krčmárová B. Krčmár M. Schwarzová M. Chlebo P. Chlebová Z. Židek R. (2018). The effects of 12-week progressive strength training on strength, functional capacity, metabolic biomarkers, and serum hormone concentrations in healthy older women: morning versus evening training. Chronobiol. Int. 35 (11), 1490–1502. 10.1080/07420528.2018.1493490 29985671

[B33] Kwon Y. H. Choi Y. W. Nam S. H. Lee M. H. (2014). The influence of time of day on static and dynamic postural control in normal adults. J. Phys. Ther. Sci. 26 (3), 409–412. 10.1589/jpts.26.409 24707094 PMC3976013

[B34] Lericollais R. Gauthier A. Bessot N. Davenne D. (2021). Diurnal evolution of cycling biomechanical parameters during a 60‐s Wingate test. Scand. J. Med. Sci. Sports 21 (6), E106–e114. 10.1111/J.1600-0838.2010.01172.X 20807387

[B35] Lopes-Silva J. P. Santos J. F. da S. Franchini E. (2019). Can caffeine supplementation reverse the effect of time of day on repeated-sprint exercise performance? Appl. Physiol. Nutr. Metab. 44 (2), 187–193. 10.1139/apnm-2018-0373 30058345

[B36] López-Samanés Á. Moreno-Pérez D. Maté-Muñoz J. L. Domínguez R. Pallarés J. G. Mora-Rodríguez R. (2016). Circadian rhythm effect on physical tennis performance in trained male players. J. Sports Sci. 35, 2121–2128. 10.1080/02640414.2016.1258481 27918240

[B37] McNair D. Lorr M. Droppleman L. (1971) EDITS manual for the profile of mood states. San Diego, CA: Editorial and Industrial Testing Service. Available at: https://cir.nii.ac.jp/crid/1573950399047802368.

[B38] Mejri M. A. Hammouda O. Yousfi N. Zouaoui K. Ben Rayana M. C. Chaouachi A. (2015). One night of partial sleep deprivation affects biomarkers of cardiac damage, but not cardiovascular and lipid profiles, in young athletes. Biol. Rhythm Res. 46 (5), 715–724. 10.1080/09291016.2015.1048951

[B39] Melhim A. F. (1993). Investigation of circadian rhythms in peak power and mean power of female physical education students. Int. J. Sports Med. 14 (06), 303–306. 10.1055/s-2007-1021182 8407059

[B40] Mhenni T. Michalsik L. B. Mejri M. A. Yousfi N. Chaouachi A. Souissi N. (2017). Morning–evening difference of team-handball-related short-term maximal physical performances in female team handball players. J. Sports Sci. 35 (9), 912–920. 10.1080/02640414.2016.1201212 27352917

[B41] Navabinejad S. Rostami M. (2023). Mind and body in sync: the fascinating field of psychophysiology in sports. Health Nexus 1 (3), 38–40. 10.61838/kman.hn.1.3.5

[B42] Oleka C. T. (2020). Use of the menstrual cycle to enhance female sports performance and decrease Sports-Related injury. J. Pediatr. Adolesc. Gynecol. 33 (2), 110–111. 10.1016/j.jpag.2019.10.002 31678355

[B43] Pullinger S. A. Oksa J. Clark L. F. Guyatt J. W. Newlove A. Burniston J. G. (2018). Diurnal variation in repeated sprint performance cannot be offset when rectal and muscle temperatures are at optimal levels (38.5°C). Chronobiol Int. 35 (8), 1054–1065. 10.1080/07420528.2018.1454938 29566344

[B44] Robinson R. H. Gribble P. A. (2008). Support for a reduction in the number of trials needed for the star excursion balance test. Arch. Phys. Med. Rehabil. 89 (2), 364–370. 10.1016/J.APMR.2007.08.139 18226664

[B45] Romero-Moraleda B. Del Coso J. Gutiérrez-Hellín J. Ruíz-Moreno C. Grgić J. Lara B. (2017). The influence of the menstrual cycle on muscle strength and power performance. J. Hum. Kinet. 68, 123–133. 10.2478/HUKIN-2019-0061 PMC6724592 31531138

[B46] Saidi O. Colin E. Rance M. Doré É. Pereira B. Duché P. (2021). Effect of morning versus evening exercise training on sleep, physical activity, fitness, fatigue and quality of life in overweight and obese adults. Chronobiol. Int. 38 (11), 1537–1548. 10.1080/07420528.2021.1935988 34128447

[B47] Sedliak M. Finni T. Peltonen J. Häkkinen K. (2008). Effect of time-of-day-specific strength training on maximum strength and EMG activity of the leg extensors in men. J. Sports Sci. 26 (10), 1005–1014. 10.1080/02640410801930150 18608836

[B48] Souissi H. Chtourou H. Chaouachi A. Dogui M. Chamari K. Souissi N. (2012). The effect of training at a specific Time-of-Day on the diurnal variations of Short-Term exercise performances in 10- to 11-Year-Old boys. Pediatr. Exerc. Sci. 24 (1), 84–99. 10.1123/PES.24.1.84 22433267

[B49] Souissi M. Souissi Y. Bayoudh A. Knechtle B. Nikolaidis P. T. Chtourou H. (2020). Effects of a 30 min nap opportunity on cognitive and short-duration high-intensity performances and mood states after a partial sleep deprivation night. J. Sports Sci. 38 (22), 2553–2561. 10.1080/02640414.2020.1793651 32734824

[B50] Souissi N. Bessot N. Chamari K. Gauthier A. Sesboüé B. Davenne D. (2007). Effect of time of day on aerobic contribution to the 30‐s Wingate Test performance. Chronobiol. Int. 24 (4), 739–748. 10.1080/07420520701535811 17701684

[B51] Souissi N. Gauthier A. Sesboüé B. Larue J. Davenne D. (2004). Circadian rhythms in two types of anaerobic cycle leg exercise: force-velocity and 30-s Wingate tests. Int. J. Sports Med. 25 (01), 14–19. 10.1055/s-2003-45226 14750007

[B52] Sutresna N. (2016). “Women athletes endurance and menstruation Cycle; pre-menstruation, 2nd day of menstruation and 5th Day of menstruation, ” in 6th international conference on educational, management, administration and leadership (Atlantis Press: Atlantis Press), 14, 334–337. 10.2991/icemal-16.2016.69

[B53] Tounsi M. Jaafar H. Aloui A. Souissi N. (2018). Soccer-related performance in eumenorrheic Tunisian high-level soccer players: effects of menstrual cycle phase and moment of day. J. Sports Med. Phys. Fit. 58 (4), 497–502. 10.23736/S02022-4707.17.06958-4 28222573

[B54] Uroh C. C. Adewunmi C. M. (2021). Psychological impact of the COVID-19 pandemic on athletes. Front. Sports Act. Living 3, 603415. 10.3389/fspor.2021.603415 33969291 PMC8096933

[B55] Valdéz P. Ramírez C. García A. (2012). Circadian rhythms in cognitive performance: implications for neuropsychological assessment. Chronophysiology Ther., 81–92. 10.2147/cpt.s32586

[B56] World Medical Association (2013). World Medical Association Declaration of Helsinki: ethical principles for medical research involving human subjects. JAMA 310 (20), 2191–2194. 10.1001/JAMA.2013.281053 24141714

